# Long-Term Use of Steroids Protects from the Development of Symptomatic Diverticulitis Requiring Hospitalization in the Asian Population

**DOI:** 10.1371/journal.pone.0124598

**Published:** 2015-04-28

**Authors:** Shen-Shong Chang, Hsiao-Yun Hu

**Affiliations:** 1 Division of Gastroenterology, Taipei City Hospital Yang-Ming Branch, Taipei, Taiwan; 2 Department of Internal Medicine, Taipei City Hospital Yang-Ming Branch, Taipei, Taiwan; 3 School of Medicine, National Yang-Ming University, Taipei, Taiwan; 4 Institute of Public Health and Department of Public Health, National Yang-Ming University, Taipei, Taiwan; 5 Department of Education and Research, Taipei City Hospital, Taipei, Taiwan; National Institute for Viral Disease Control and Prevention, CDC, China, CHINA

## Abstract

**Objective:**

The pathophysiology of diverticulitis is poorly understood. Factors such as physical inactivity, constipation, obesity, smoking, and the use of nonsteroidal antiinflammatory drugs (NSAIDs) have been associated with an increased risk of diverticular disease. To evaluate whether patients exhibiting long-term steroid use are at increased risk of colonic diverticulitis.

**Method:**

We conducted a population-based, nested case–control study. Data were retrospectively collected from the National Health Insurance Research Database. The study cohort comprised patients diagnosed with diverticulitis, identified using inpatient discharge records using International Classification of Diseases, Ninth Revision, Clinical Modification (ICD-9-CM) codes (562.11 and 562.13), and those who were administered one or more prescriptions for corticosteroids for systemic use. Control patients were matched to cases by age, sex, NSAID use, laxative drug use, and index date. We enrolled 690 patients with colonic diverticulitis and 2760 in the control group. The adjusted odds ratios (ORs) and 95% confidence intervals (CIs) were estimated using conditional logistic regression.

**Results:**

Compared with steroid nonusers, the adjusted ORs were 0.60 (95% CI = 0.35–1.06) and 0.80 (95% CI = 0.64–1.008) in current steroid users and previous steroid users, respectively. In addition, the adjusted ORs were 0.55 (95% CI = 0.31–0.98), 0.57 (95% CI = 0.31–0.98), and 0.44 (95% CI = 0.22–0.86) for steroid use duration more than half time by an exposure period of 90 days, 180 days, and 365 days before the claim date of colonic diverticulitis, respectively.

**Conclusions:**

The results indicated that long-term steroid use within one year is associated with lower risk of colonic diverticulitis.

## Introduction

Colonic diverticulosis is an acquired disease, developing as mucosal and submucosal herniations through the circular muscular layer [1,2] and appears to be increasing in both asymptomatic and symptomatic presentations [2] Only 10%–20% of patients with diverticulosis develop diverticulitis [3,4]. Stasis or obstruction in the narrow-necked diverticulum can lead to bacterial overgrowth [5].

The pathophysiology of diverticulitis is poorly understood. Factors such as physical inactivity, constipation, obesity, smoking, and the use of nonsteroidal antiinflammatory drugs (NSAIDs) have been associated with an increased risk of diverticular disease [6]. Strate et al [7] observed that regular use of aspirin or NSAIDs is associated with an increased risk of diverticulitis, and proposed that mechanisms such as direct injury or impaired prostaglandin synthesis increase permeability and the influx of bacteria and other toxins [8]. Von Rahden et al [9] indicated a strong correlation of glucocorticoid-induced tumor necrosis factor receptors with matrix metalloproteinase-9 in diverticulitis, which might support the functional connection of these molecules with complicated sigmoid diverticulitis. Humes et al [10] showed that steroids and opiate analgesics drugs were strongly associated with an increased risk of diverticular perforation in western societies.

Steroid intake has been identified as a crucial risk factor leading to a complicated type of sigmoid diverticulitis [11]. By contrast, corticosteroids are the most effective antiinflammatory therapy for numerous chronic inflammatory diseases such as asthma and inflammatory bowel disease (IBD). We conducted a nested case-control study based on the National Health Insurance Research Database (NHIRD) in Taiwan. The primary objective was to assess the association between steroid use and the occurrence of colonic diverticulitis in Asian societies.

## Materials and Methods

### Ethical considerations

NHIRD is a secondary database. The information on the identity of subjects from the database was scrambled before it was released for research purpose. The privacy and confidentiality of all beneficiaries were safeguarded by the Taiwan National Health Research Institute (NHRI). The data is publicly available. Patient records/information was anonymized and de-identified prior to analysis. In this study, ethics approval was approved by the NHRI and the Institutional Review Board (IRB) of Taipei City Hospital (IRB No.: TCHIRB-1021103-E). Written consent was waived by the approving IRB.

### Data Source

This nationwide cohort study was based on patient data obtained from the NHIRD, which is managed by the Taiwanese NHRI. The NHIRD contains health care data for 99% of the Taiwan population (approximately 23 million people) [12]. The NHIRD sample files are composed of comprehensive use and enrollment information for a randomly selected sample of 1 000 000 National Health Insurance (NHI) beneficiaries, representing approximately 5% of all enrollees in the year 2000. We used the International Classifications of Diseases, Ninth Revision, Clinical Modification (ICD-9-CM) to define diseases.

### Identifying Cases and Control

**Fig 1:**
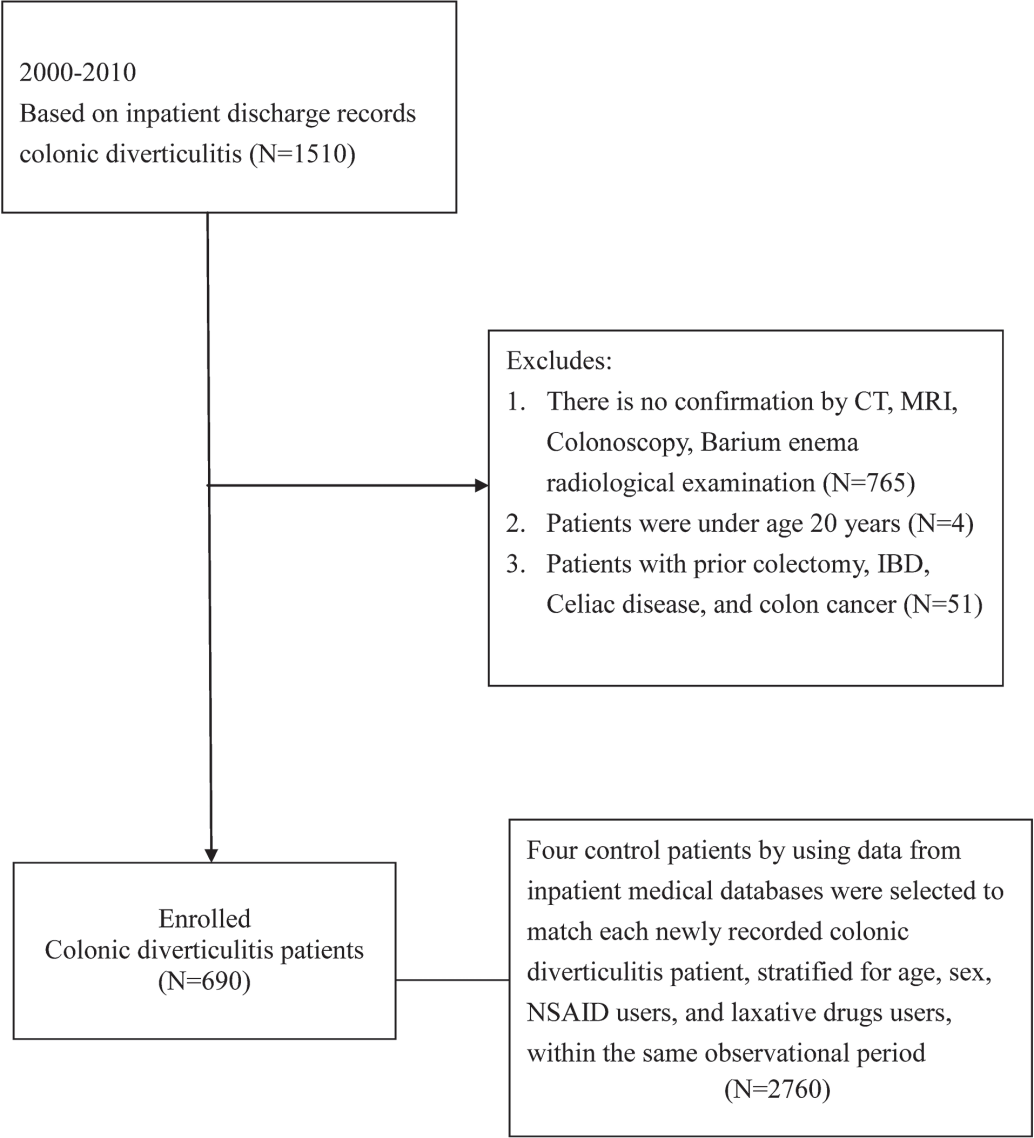
Flow Chart Depicting the Selection of the Participants.

Diverticulitis patients were identified from the NHIRD by using inpatient discharge records based on ICD-9-CM codes (562.11 and 562.13) following computed tomography (CT), magnetic resonance imaging (MRI), colonoscopy, and confirmed barium radiological examination between January 1, 2000 and December 31, 2010. Patients with a prior colectomy, celiac disease, and IBD were excluded. We also excluded patients diagnosed with colon cancer between 1 January, 1996 and the index date. Patients under the age of 20 years were excluded. To be defined as the control group, a patient could not have code ICD-9-CM: 562.xx in their inpatient records or in the ambulatory care claims. Using data from inpatient medical databases, 4 control patients were selected to match each newly recorded colonic diverticulitis case by using random sampling, and control patients were stratified for age, sex, NSAID use, and laxative drugs use from the database within the same observation period. Fig 1 show a flow chart containing the total patients included.

### Determining Steroid Medication Exposure

#### Drug Exposure Data

Information on all steroids (including oral prednisolone, dexamethasone, betamethasone, triamcinolone, and fludrocortisones) prescription was extracted from the NHRI prescription database. Cumulative steroid use duration was estimated as the sum of days of drugs dispensed by an exposure time of 90 days, 180 days, and 365 days before the claim date of hospitalization for colonic diverticulitis, respectively. We defined current steroid users as those with steroid exposure lasting 28 days before the claim date of hospitalization for colonic diverticulitis. We defined previous users as those with steroid exposure between January 1, 2000 and the claim date of hospitalization for colonic diverticulitis, excluding current users. We defined nonusers as those without a prescription for steroids at any time between January 1, 2000 and the claim date of hospitalization for colonic diverticulitis. In addition, the defined daily dose (DDD) is the assumed average maintenance dose per day for drugs administered to adults and used according for their main indications. The DDD recommended by the World Health Organization (WHO) [13] was used to quantify the use of steroids in this study. Cumulative DDD was estimated as the sum of the dispensed DDD of any steroid use from 1 year before the index date. The research data comprised the date of prescription, daily dose, and the number of days of medication.

### Assessing Cofounders and Covariates

#### Comorbid Illness

Conditions that required inpatient care between January 1, 1996, and the index date of our study were defined as comorbidities. Steroids are used to treat systemic diseases, such as respiratory disease, rheumatological disease, allergy disease, dermatitis, renal disease, endocrine disease, neurological disease, and hematological tumors. The comorbidities identified in our cohort and the corresponding ICD-9-CM diagnosis codes were as follows: respiratory disease, including asthma (ICD-9-CM Code 493), chronic obstructive pulmonary disease (COPD; ICD-9-CM Codes 490–492, 494, and 496), and sarcoidosis (ICD-9-CM Code 135); rheumatological disease, including diseases of the musculoskeletal system and connective tissue (ICD-9-CM Codes 710,712,714, and 720–724) and vasculitis (ICD-9-CM Codes 446–447); allergy disease (ICD-9-CM Codes 995.3 and 477); dermatitis (ICD-9-CM Codes 690–698); renal disease, including glomerulonephritis and nephrotic syndrome (ICD-9-CM Codes 580–582); endocrine disease, such as disorders of the adrenal glands (ICD-9-CM Code 255); neurological disease, including central nervous system (CNS) tumors (ICD-9-CM Codes 191–192,225) and cerebral edema (ICD-9-CM Code 348.5); and hematological tumors (ICD-9-CM Codes 200–208).

### Use of Other Medications

Patients were defined as users of NSAIDs, laxative drugs, aspirin, cyclooxygenase-2 (COX-2) specific inhibitors, and antidiarrhea drugs based on whether they had at least one prescription of the respective medication within 28 days of the claim date of hospitalization for colonic diverticulitis.

### Statistical Analysis

For comparisons of proportions, chi-square statistics were used. A conditional logistic regression model was used to estimate the relative magnitude related to the use of steroids. Exposure was defined as patients who received steroids between January 1, 2000 and the index date. In the analysis, the patients were categorized into one of 3 steroid exposure categories: nonusers, previous users, and current users. The studied participants were categorized based on whether they received steroid doses below the median (< 11.25 DDD) or equal and above than median (≥ 11.25 DDD). In the dose–response analysis, we calculated the odds ratios (OR) for the above-the-median group (≥ 11.25 DDD) and the below-the-median group (< 11.25 DDD) doses, and for the cumulative treatment duration within 1 year. ORs and their 95% confidence intervals (CIs) were calculated using patients with no exposure as the reference. Models with and without adjusted variables are presented. All statistical analyses were performed using the SAS System for Windows, Version 9.3 (SAS Institute, Cary, NC, USA).

## Results

We enrolled 690 patients diagnosed with colonic diverticulitis and 2760 control group patients from 2000 to 2010. Table 1 lists the demographic data, including age, sex, comorbidities, medications, and the number of hospital days. The patients diagnosed with colonic diverticulitis exhibited a markedly lower rate of aspirin use and COX-2 inhibitor use, but a higher rate of antidiarrhea drug use. The number of hospital days was 11.73 ± 53.52, 9.88 ± 11.29, 26.01 ± 24.83, and 9.80 ± 12.92 days for the steroid users, nonusers, current users, and previous users, respectively.

**Table 1:** Characteristics of subjects admitted with acute diverticulitis and matched controls.

Variables	Control		Diverticulitis		*p* value
	n = 2760	%	n = 690	%	
Sex					1.000
Male	1492	54.06	373	54.06	
Female	1268	45.94	317	45.94	
Age					1.000
20–49	844	30.58	211	30.58	
50–69	944	34.20	236	34.20	
≥70	972	35.22	243	35.22	
Comorbidities					
Respiratory disease	283	10.25	69	10.00	0.844
Rheumatological disease	188	6.81	50	7.25	0.687
Allergic disease	13	0.47	3	0.43	0.900
Dermatitis	50	1.81	10	1.45	0.515
Renal disease	72	2.61	20	2.90	0.673
Endocrine disease	23	0.83	9	1.30	0.248
Neurological disease	21	0.76	3	0.43	0.357
Hematological tumor	17	0.62	3	0.43	0.575
Medication use					
NSAIDs	808	29.28	202	29.28	1.000
Laxative drugs	1056	38.26	264	38.26	1.000
Aspirin	322	11.67	58	8.41	0.014
COX-2 inhibitors	176	6.38	30	4.35	0.044
Anti-diarrhea drugs	200	7.25	84	12.17	<0.001
Steroid use					0.323
Non-users	1219	44.17	307	44.49	
Current use	128	4.64	23	3.33	
Previous use	1413	51.20	360	52.17	
Hospital days			11.73±53.52		
Non-users	-		9.88±11.29		
Current users	-		26.01±24.83		
Previous users	-		9.80±12.92		

Respiratory disease including asthma, chronic obstructive pulmonary disease, and sarcoidosis; Rheumatological disease including diseases of the musculoskeletal system and connective tissue, and vasculitis; Renal disease including glomerulonephritis and nephrotic syndrome; Endocrine disease including disorders of adrenal glands; Neurological disease including central nervous system tumor and cerebral edema; NSAIDs: non-steroidal anti-inflammatory drugs. COX-2 inhibitors: cyclooxygenase-2 specific inhibitors, N: number.

Compared with the steroid nonusers, the adjusted ORs were 0.60 (95% CI = 0.35–1.06) and 0.80 (95% CI = 0.64–1.008) for current steroid users and previous steroid users, respectively (Table 2). In addition, compared with steroid nonusers, the adjusted ORs were 1.07 (95% CI = 0.75–1.51), 1.26 (95% CI = 0.95–1.66), and 1.09 (95% CI = 0.86–1.38) for steroid use duration less than half time (45 days, 90 days, 180 days, respectively) by an exposure time of 90 days, 180 days, and 365 days before the claim date of hospitalization for colonic diverticulitis, respectively. The adjusted ORs were 0.55 (95% CI = 0.31–0.98), 0.57 (95% CI = 0.31–0.98), and 0.44 (95% CI = 0.22–0.86) for the steroid use duration more than half time by an exposure period of 90 days, 180 days, and 365 days before the claim date of hospitalization for colonic diverticulitis, respectively (Table 2).

**Table 2:** Association between exposure to steroids (duration) and acute diverticulitis.

Exposure	Cases Exposed/Unexposed	Controls, Exposed/Unexposed	Crude OR (95%CI)	Adjusted OR* (95%CI)
Current Use	23/667	128/2632	0.70 (0.45–1.11)	0.60 (0.35–1.06)
Previous Use	384/284	1493/1138	1.03 (0.86–1.24)	0.80 (0.64–1.00)
Steroids use within 90days preceding index date				
duration < 45days	71/619	248/2512	1.16 (0.87–1.54)	1.07 (0.75–1.51)
duration ≧ 45 days	20/670	108/2652	0.75 (0.46–1.21)	0.55 (0.31–0.98)
Steroids use within 180days preceding index date				
duration < 90 days	120/570	391/2369	1.28 (1.01–1.60)	1.26 (0.95–1.66)
duration ≧ 90 days	18/672	90/2670	0.83 (0.50–1.39)	0.57 (0.31–0.98)*
Steroids use within 365days preceding index date				
duration < 180 days	177/513	619/2141	1.19 (0.98–1.45)	1.09 (0.86–1.38)
duration≧ 180 days	16/674	84/2676	0.79 (0.46–1.36)	0.44 (0.22–0.86)

*Adjusted for matching variables such as comorbidities (including respiratory disease, rheumatological disease, allergy disease, dermatitis, renal disease, endocrine disease, neurological disease, and hematological tumor) and medications (including NSAIDs, laxative drugs, aspirin, COX-2 inhibitors, and anti-diarrhea drugs)

Steroid use was categorized according to the cumulative dose, and the model was adjusted for potential confounders. Compared with the steroid nonusers, the adjusted ORs were 1.31 (95% CI = 0.98–1.74), 1.02 (95% CI = 0.73–1.40), 1.65 (95% CI = 0.72–3.78), and 1.35 (95% CI = 0.82–2.23) for the cumulative steroid doses < 11.25 DDD, prednisolone doses < 10.5 DDD, betamethasone doses < 13 DDD, and dexamethasone doses < 4.75 DDD, respectively. The adjusted ORs were 0.71 (95% CI = 0.51–0.99), 0.76 (95% CI = 0.53–1.08), 0.65 (95% CI = 0.26–1.59), and 0.77 (95% CI = 0.42–1.41) for the cumulative steroid doses ≥ 11.25 DDD, prednisolone doses ≥ 10.5 DDD, betamethasone doses ≥ 13 DDD, and dexamethasone doses ≥ 4.75 DDD, respectively (Table 3).

**Table 3:** Association between exposure to steroids (dosage) and acute diverticulitis.

Exposure	Cases Exposed/Unexposed	Controls, Exposed/Unexposed	Crude OR (95%CI)	Adjusted OR* (95%CI)
Steroids use				
DDD < 11.25	112/578	347/2413	1.34 (1.06–1.71)	1.31 (0.98–1.74)
DDD ≧ 11.25	81/609	356/2404	0.94 (0.72–1.23)	0.71 (0.51–0.99)
Prednisolone use				
DDD < 10.5	78/612	272/2488	1.16 (0.88–1.52)	1.02 (0.73–1.40)
DDD ≧ 10.5	65/625	272/2488	0.97 (0.73–1.30)	0.76 (0.53–1.08)
Betamethasone use				
DDD < 13	15/675	27/2733	2.21 (1.18–4.18)	1.65 (0.72–3.78)
DDD ≧ 13	8/682	34/2726	0.95 (0.44–2.06)	0.65 (0.26–1.59)
Dexamethasone use				
DDD < 4.75	27/663	90/2670	1.20 (0.78–1.86)	1.35 (0.82–2.23)
DDD ≧ 4.75	21/669	96/2664	0.87 (0.54–1.41)	0.77 (0.42–1.41)

*Adjusted for matching variables such as comorbidities and medications.

The number of patients using triamcinolone and fludrocortisones was too small to calculate.

Moreover, compared with the steroid nonusers, after adjustment for possible confounders, the adjusted ORs were 0.29 (95% CI = 0.02–5.25), 0.31 (95% CI = 0.02–5.55), and 0.27 (95% CI = 0.02–4.66) for the steroid use duration more than half time by an exposure period of 90, 180, and 365 days before the claim date of hospitalization for colonic diverticulitis requiring surgery, respectively. In addition, no significant trend was observed in decreasing colonic diverticulitis requiring surgery with increasing cumulative steroid doses ≥11.25 DDD (adjusted OR = 0.71, 95% CI = 0.20–2.52) within 365 days before the claim date, as shown in Table 4.

**Table 4:** Association between exposure to steroids (duration and dose) and acute diverticulitis requiring surgery (*N* = 320).

Exposure	Cases Exposed/Unexposed	Controls, Exposed/Unexposed	Crude OR (95%CI)	Adjusted OR* (95%CI)
Steroids use within 90days preceding index date				
duration < 45days	6/58	26/230	0.90 (0.34–2.40)	0.88 (0.23–3.37)
duration ≧ 45 days	2/62	10/246	0.79 (0.17–3.70)	0.29 (0.02–5.25)
Steroids use within 180days preceding index date				
duration < 90 days	11/53	46/210	0.96 (0.46–2.00)	0.83 (0.29–2.36)
duration ≧ 90 days	2/62	5/251	1.59 (0.31–8.22)	0.31 (0.02–5.55)
Steroids use within 365days preceding index date				
duration < 180 days	12/52	69/187	0.61 (0.29–1.24)	0.48 (0.17–1.33)
duration ≧ 180 days	2/62	5/251	1.44 (0.28–7.44)	0.27 (0.02–4.66)
Steroids use within 365days preceding index date				
DDD < 11.25	5/59	40/216	0.42 (0.15–1.17)	0.28 (0.07–1.17)
DDD≧ 11.25	9/55	34/222	0.96 (0.42–2.19)	0.71 (0.20–2.52)

*Adjusted for matching variables such as comorbidities and medications.

*N* = number

## Discussion

The results of this study indicate that patients who are long-term steroid users have a reduced risk of developing colonic diverticulitis (OR = 0.44, 95% CI = 0.22–0.86, *p* = 0.016). However, an increased mean daily dose of steroids was weak negative associated with a developing colonic diverticulitis (OR = 0.71, 95% CI = 0.51–0.99, *p* = 0.044) within 365 days before the claim date. Factors such as constipation and NSAIDs have been associated with an increased risk of colonic diverticulitis [6]. Therefore, 4 control patients were selected to match colonic diverticulitis case stratified by NSAID use and laxative drugs use.

A study conducted by von Rahden et al [9] enrolled only 22 patients exhibiting steroid treatment, most of whom (19/22) belonged to surgical complicated diverticulitis. Patients in this group received surgery for complicated diverticulitis, such as covered perforation (73.7%), free perforation (21%), and phlegmonous diverticulitis (5.3%). Chapman et al [11] conducted a 13-year retrospective study in a single hospital with a chart review and disclosed that steroid use was significantly associated with perforation rates and mortality in complicated diverticulitis patients. This outcome differs from that of the present study. However, our methodology differed from that of Chapman et al [11]. The previous study enrolled patients with complicated diverticulitis with perforation, abscess, obstruction, phlegmon, fistula, or bleeding, and were treated with surgical intervention. Among them, 74 patients (22%) were using steroids at the time of the complicated diverticulitis episode (duration unknown and only as a cofactor). However, our methods included analyzing the association among steroid use duration, dose–response, and symptomatic colonic diverticulitis. This study, which was a nationally-based, nested case–control, 11-year longitudinal study, focused on the steroid use dose/duration of colonic diverticulitis patients (only 64 patients received surgical intervention).

NSAIDs have been implicated as a possible factor contributing to bleeding and perforation from diverticulitis [7,14,15]. These drugs reduce gut prostaglandin synthesis, which affects the maintenance of the mucosal barrier and results in mucosal damage, increased colonic permeability, and bacterial translocation [16]. Humes et al [10] disclosed that perforated diverticular disease is a severe surgical emergency, and current opiate analgesics and oral corticosteroids (OR = 2.74; 95% CI = 1.63–4.61) are strongly associated with an increased risk of perforation. By contrast, our data suggested that steroid use duration-response is a factor that reduces acute diverticulitis, which is dissimilar to the result of Humes et al. The primary discrepancy is the enrolled patient criteria. Only 9.28% (*n* = 320) of the colonic diverticulitis patients received surgical intervention, which is dissimilar to a study conducted by Humes.

No increased risk was observed for developing colonic diverticulitis requiring surgery in long-term or high-dose steroid users in this study. The results of this study show that the risk of developing episodes is lower, although this did not reach statistical significance, which conflicts with studies conducted in Western countries. Performing a prospective randomized controlled study facilitated an evaluation of whether steroid use—including the duration they are used and the prescribed dose—can protect patients against the onset of diverticulitis; however, once diverticulitis has been diagnosed, it is more severe in steroid users in Asia. The consequences of diverticulitis might be more severe in immunocompromised patients. Corticosteroids are the most effective antiinflammatory therapy for numerous chronic inflammatory diseases. Further study is required to elucidate the role of corticosteroids, including duration and dose in immune-competent and immunocompromised patients, diagnosed with varying extents of diverticulitis.

Certain data, such as the location of diverticulitis, are unavailable in the NHIRD. Left-sided diverticular disease is most common in western societies, whereas right-sided diverticular disease is more prevalent in Asia and Africa. In addition, compared with western societies, colonic diverticular disease is uncommon in Asian societies. The data from the Taiwan NHI revealed highly similar distributions of diverticulitis patients in the 20–49 (30.58%), 50–69 (34.20%), and ≥ 70-year-old age groups (35.22%), indicating a distinct mechanism of disease pathogenesis compared to Western populations, where most patients are elderly adults. Our data require caution when extrapolating to western societies because of diverse lifestyles. Further study is required to confirm whether mostly right-sided colonic diverticulitis in Asia may be a different entity.

Establishing a standard definition is crucial; therefore, we analyzed only the hospitalization for symptomatic colonic diverticulitis by using data from inpatient medical databases. The true prevalence of diverticulosis is difficult to define because most people are asymptomatic. A routine colonoscopy or barium radiological examination is not reimbursed in the Taiwanese NHI program. Therefore, we conducted this study using a nested case–control method instead of a cohort study.

Diverticulitis status might not be constant in the same patients because diverticulitis might improve after they recover from an acute illness. The clinical process of colonic diverticulitis vary in accordance with the extent of the disease progress. Because only 10%–20% of patients with diverticulosis develop diverticulitis, proving that these patients have recurrent asymptomatic diverticulitis might be difficult. Therefore, we adopted a strict definition of the control group, in which a patient cannot exhibit ICD-9-CM code 562.xx in their inpatient records or in the ambulatory care claims between 2000 and 2010. To be included in the study group, patients were identified using ICD-9-CM codes and following CT, MRI, colonoscopy, and barium radiological image examination (excluding 765 cases without image examination). Although imaging reports are unavailable in the NHIRD, the Bureau of the NHI randomly samples the claims data from every hospital and reviews charts to verify diagnostic validity. ICD-9-CM coding was strictly audited for the purpose of reimbursement.

This study has several limitations. First, the observations were based on hospitalization for symptomatic colonic diverticulitis. Caution must be used in extrapolating these results to other population characteristics, such as asymptomatic diverticulosis and peritonitis with septic perforated diverticulitis. Second, although we conducted a multivariate analysis, numerous factors were unavailable for adjustment, such as patients’ lifestyle, dietary habits (particularly fiber intake) [17], and body mass index [18]. In addition, the site of diverticulitis is not available from the NHIRD. In Asia, 80% of diverticulitis cases occur on the right side, which is the opposite of cases reported in most Western countries. To confirm the association between steroid dose/duration and symptomatic diverticulitis in Asian populations, a well-designed prospective cohort study is warranted. Such a study should consider the location of diverticulitis and obtain information on the dietary, smoking, and drinking habits of patients. Third, the consumption of steroids could not be confirmed in this study. However, the Bureau of the NHI has formed various audit committees that randomly sample claims data from every hospital and review charts on a regular basis to verify the diagnostic validity and quality of care. Physicians seldom prescribe long-term steroids dosages for patients, particularly those who have contracted chronic illnesses, if the patients have not taken these medications. Moreover, whether patients used over-the-counter oral steroid medications was not included in our research data. Fourth, steroid use may have masked the symptoms of diverticulitis. However, we obtained NHI data from 1 year before the index date. This period is sufficient for complications such as perforation, bleeding, or symptomatic diverticulitis to manifest—even if the symptoms are masked. Therefore, the study period was considered adequate to ensure that diverticulitis patients were not overlooked. Finally, because we did not have data regarding the severity of diverticulitis graded according to Hinchey’s criteria, we considered patients based on whether they had diverticulitis requiring surgery, rather than evaluate each case based on the severity of their condition.

In conclusion, our results provide evidence to support the view that long-term steroid use within one year is associated with lower risk of colonic diverticulitis in Asian societies.
